# Ovarian microcystic stromal tumor with omental metastasis: the first case report and literature review

**DOI:** 10.1186/s13048-021-00812-1

**Published:** 2021-05-27

**Authors:** Xiaxia Man, Zhentong Wei, Baogang Wang, Wanying Li, Lingling Tong, Liang Guo, Songling Zhang

**Affiliations:** 1 Department of Oncologic Gynecology, The First Hospital of Jilin University, Xinmin Street 1,Changchun, Jilin, 130021 People’s Republic of China; 2 Department of Cardiac Surgery, The First Hospital of Jilin University, Changchun, 130021 Jilin, People’s Republic of China; 3 Department of Pathology, the First Hospital of Jilin University, Xinmin Street 1,Changchun, 130021 Jilin, People’s Republic of China

**Keywords:** β-Catenin, Microcystic stromal tumor, Ovary, Immunophenotype, Metastasis

## Abstract

**Background:**

Microcystic stromal tumor (MCST) of the ovary is an extremely rare subtype of sex cord-stromal neoplasm first described by Irving and Young in 2009. Tumors from all previously reported cases (fewer than 40 total) were benign, but one was a case of ovarian MCST that reoccurred.

**Case presentation:**

Herein, we present a unique single case of ovarian MCST with omental metastasis in a 47-year-old Chinese female along with its histologic and immunohistochemical profile and genetic alterations. The tumor exhibited the previously described classic microscopic features and immunoprofiles of MCST. The tumorlet in the omentum presented the same histological structures and characteristically expressed β-catenin protein (localized in the nucleus). Molecular analysis identified a point mutation (c.98C > G) in exon 3 of *CTNNB1*.

**Conclusions:**

To the best of our knowledge, no such report has been documented for ovarian MCST with omental metastasis. The study may provide new insights into the tumor biology of MCST and provide a better understanding of this rare entity.

**Supplementary Information:**

The online version contains supplementary material available at 10.1186/s13048-021-00812-1.

## Introduction

In 2009, Irving and Young originally described a new distinct histopathologic subtype of neoplasm called microcystic stromal tumor (MCST) of the ovary [1]. The distinctive histological characteristics include microcystic, solid cellular regions and a hyalinized fibrous stroma, with immunohistological features of diffuse and strong positive staining for CD10 and β-catenin (localized in the nucleus). Genetically, alterations in the CTNNB1 gene or in other genes involved in the Wnt/β-catenin pathway are involved in MCST tumorigenesis. Previously, all cases of MCST worldwide were described as having benign biological behavior, but one case of ovarian MCST that presented with recurrence has been identified [2]. Recently, we encountered a case of ovarian MCST that exhibited features similar to those reported, but omental metastasis was unexpectedly identified in the postoperative histopathological specimen. This was the first case of MCST with omental metastasis, which indicates undetermined potential or even malignant biological actions.

## Case presentation

The study was approved by the Institutional Review Board of The First Hospital, Jilin University (IRB No. 2019–302) and performed in accordance with the principles of the Declaration of Helsinki. Written informed consent was obtained from the patient for publication of this case report and any accompanying images.

**Fig. 1:**
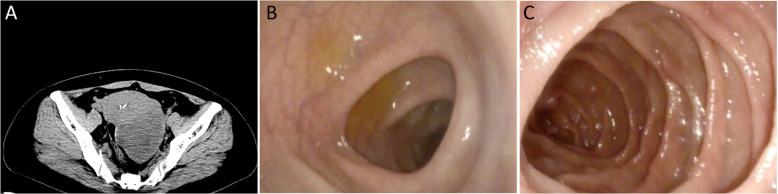
Clinical evaluation of the patient. **a** Image of the tumor. Computed tomography showed an 89 × 68 mm solid-cystic mass in the left ovary. **b** Image from the colonoscopy. The colonic mucosa was smooth, and no nodules or polyps were found. **c** Image from the gastroscopy. The gastric mucosa is smooth, and no nodules or polyps are found

A 47-year-old Chinese female, gravida 1, para 1 (G1P1) with no pertinent past medical history, was admitted to our hospital because of abdominal discomfort for 1 mo. Physical examination revealed a solid and cystic mobile mass in the left adnexal regions. Abdominal CT imaging revealed a 89 × 68 mm sized left ovarian mass with solid and cystic portions, which raised the possibility of a sex cord stromal tumor, and it was suspected to be a malignant epithelial tumor (Fig. 1a). Preoperative serum CA-125, CA-199 and carcinoembryonic antigen levels were normal. Given this mass, the patient underwent laparotomy and left salpingo-oophorectomy. At operation, the mass had a bosselated, smooth surface without obvious evidence of peritoneal involvement. The result of intraoperative frozen biopsy was a sex cord stromal tumor, and a granulosa cell tumor could not be excluded. Accordingly, staging laparotomy, including hysterectomy, bilateral salpingo-oophorectomy, bilateral pelvic lymphadenectomy, omentectomy and staging biopsies, was performed. After the immunohistochemical examination, the patient was finally diagnosed with MCST, and the pelvic lymph nodes were free from tumors. However, an unexpected typical omental metastasis was confirmed according to randomized omentum pathological sections. Because of the possibility of familial adenomatous polyposis (FAP), the patient was referred for combined upper (Fig. 1b) and lower (Fig. 1c) gastrointestinal endoscopy. Eventually, FAP was ruled out. No further oncologic therapy was administered. She is currently disease-free at 10 months postoperation and is scheduled for follow-up after 19 months.

### Pathologic findings

**Fig. 2:**
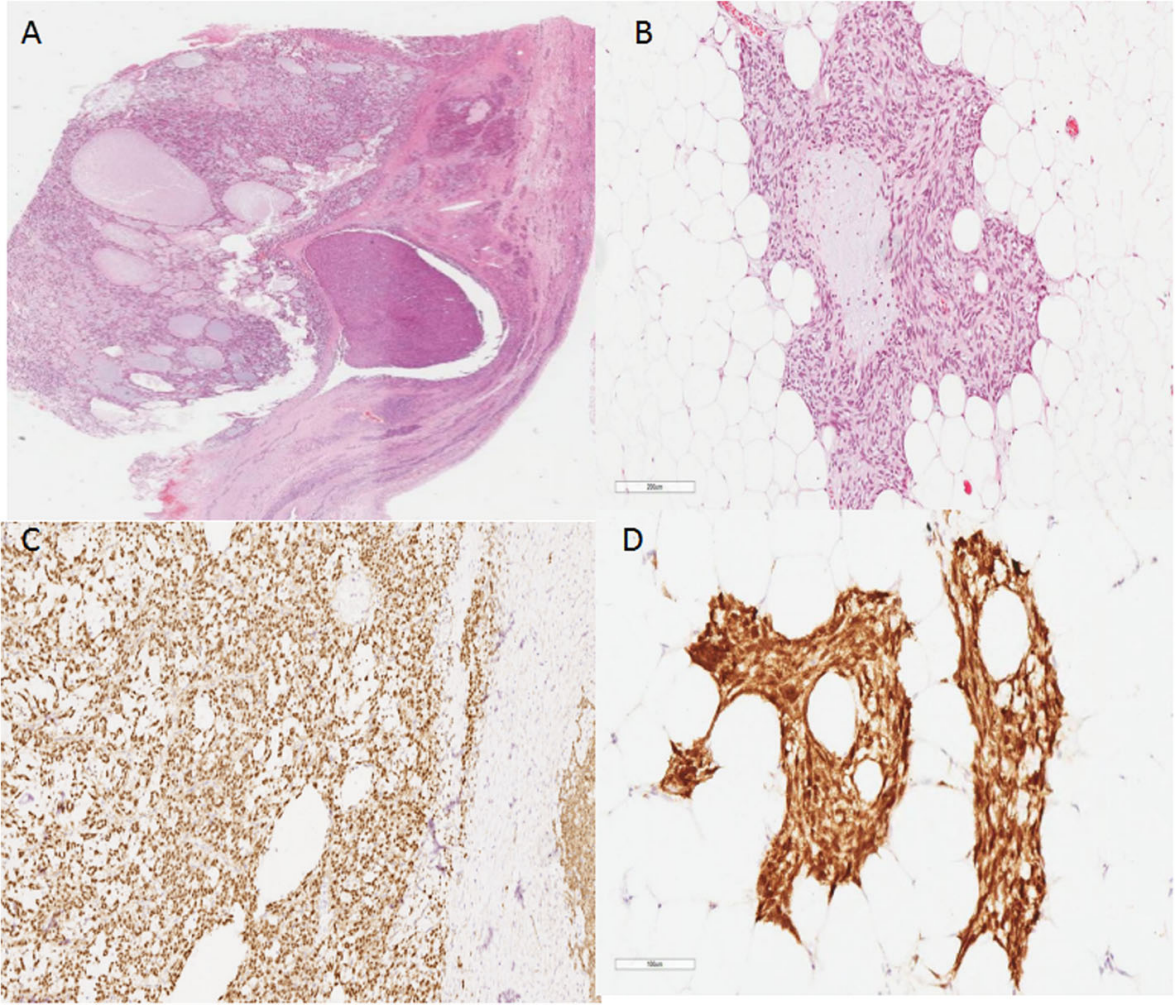
Histology and immunohistochemistry of the tumor. **a** Whole slide scan showing a cystic growth pattern with a partial solid area. H&E, × 4 magnification. **b** Tumorlet in the omentum. H&E, × 200 magnification. **c** Positive immunohistochemical staining for β-catenin (nuclear and cytoplasmic). The tumor cells are nuclear and cytoplasmic positive while the normal ovarian stromal cells on the right margin of the graph are positive on the membrane. Envision × 100 magnification. **d** The tumorlet in the omentum was positive for β-catenin (nuclear and cytoplasmic). Envision × 200 magnification

A nodular mass measuring 95 × 65 × 58 mm with an intact capsule was sent to the pathology department at our hospital. The cut surface was solid, soft and tan to gray in color. There were small multiple cystic changes in the focal areas of the tumor with gelatinous material. In the low power field view, the tumor was predominantly microcystic with a partial/focal solid region (Fig. 2a). Under higher magnification, the solid area was composed of medium-sized tumor cells with eosinophilic cytoplasm. The tumor was separated by fibers with hyalinization. Mitosis was scarce. Small nucleoli and intracytoplasmic vacuoles could be seen. There was a tumorlet (2 mm in diameter) in the omentum with the same microcystic structure and histologic features of tumor cells as the left ovary (Fig. 2b).

### Immunohistochemical studies

Surgical specimens were fixed in 10% neutral-buffered formalin and routinely processed. Paraffin-embedded blocks were sectioned (3 mm-thick) and stained with hematoxylin and eosin. Immunohistochemistry was performed using paraffin-embedded tissue samples using the streptavidin-peroxidase method. Primary antibodies were purchased from GSGB-BIO (Beijing, China) and Maxvision (Fuzhou, China) and used according to the manufacturer’s instructions.

The sex cord tumor markers calretinin and inhibin-α were both negative; however, CD10 and β-catenin (nuclear and cytoplasmic) (Fig. 2c) were both positive. The other positive markers were WT-1, SF-1, cyclin D1, AR, ER, PR, vimentin, and CD99 (perinuclear dot-like). Focal areas of the tumor were positive for CK7, CK-pan, CD56, Syn, and SMA. The tumor was negative for SALL4, CD34, and E-cadherin. The Ki-67 index was very low, and MMR protein expression was intact (Supplementary Fig. 1). The reticular fibers around individual tumor cells were identified by reticulin staining. The tumorlet in the omentum was positive for β-catenin (nuclear and cytoplasmic) (Fig. 2d).

### Molecular studies

Genomic DNA was extracted from 5 mm-thick unstained sections cut from formalin-fixed paraffin-embedded tumor blocks using an Ezup Column Animal Tissue Genomic DNA Extraction Kit (B518251, Sangon Biotech, Shanghai, China) according to the manufacturer’s instructions. Exon 3 of *CTNNB1* was amplified by PCR using the following specific primer pairs: 5′-GATTTGATGGAGTTGGACATGG-3′ (sense) and 5′-GCTACTTGTTCTTGAGTGAAGG-3′ (antisense). The PCR products were confirmed by agarose gel electrophoresis, purified using the DNA Clean/Extraction Kit (B518141, Sangon Biotech, Shanghai, China), and submitted for direct sequencing using BigDye Terminator v1.1 (Applied Biosystems, Carlsbad, CA, USA) according to the manufacturer’s protocol. The sequencing products were ethanol-precipitated before running on a 3730XL Genetic Analyzer (Applied Biosystems, Foster City, CA, USA), and the resulting sequence data were analyzed using Chromas software. Each mutation was verified in both the sense and antisense directions and was evaluated independently by two investigators.

**Fig. 3:**
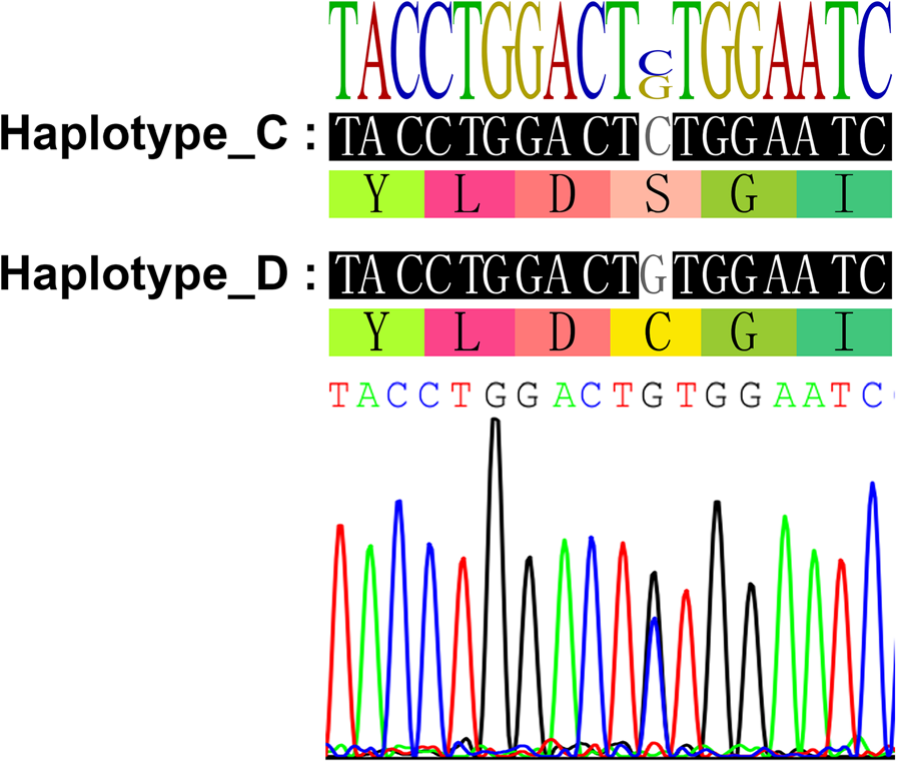
Molecular analysis of the tumor. Sequence chromatogram of the case harboring a point mutation in exon 3 of *CTNNB1* (c.98C > G)

DNA sequencing analysis revealed a missense mutation, c.98C > G, in exon 3 of *CTNNB1* (Fig. 3), which caused the replacement of serine with cysteine (UCU > UGU) at codon 33 and led to the loss of the glycogen synthase kinase (GSK)-3β phosphorylation site in β-catenin.

## Discussion

Ovarian MCST is a rare variant of the pure stromal tumor that was first described by Irving and Young in 2009 [1]. Additional studies have been published since then, and demonstrated the unique histologic and immunohistochemical features of MCST, including the involvement of the Wnt/β-catenin pathway in the pathogenesis. Nevertheless, to date, less than 40 cases of MCST have been reported worldwide, all of which are described as having benign biological behavior, but one patient experienced recurrence [1–14].

The tumor of the present case includes all classical histologic features of MCST, such as microcysts, solid cellular regions, and hyalinized fibrous stroma. The markers of sex cord tumors (calretinin and inhibin-α) and germ cell tumors (SALL4 and OCT3/4) are negative, while CD10 and β-catenin (nuclear and cytoplasmic) are characteristically positive. Other positive markers reported in previous literature, such as WT-1, SF-1, cyclin D1, AR, ER, PR, vimentin, and CD99 (perinuclear dot-like), are also expressed in our case. The expression profiles of relative markers from references listed in present article are summarized in Table 1. Both morphology and immunoprofile exclude other microcystic tumors of the ovary but MCST. Tumors in the omentum share the same histologic features and β-catenin immunoexpression as those in the left ovary, which is highly indicative of omentum metastasis. Mitosis is rare, and the Ki-67 index is low, indicating a low proliferation rate of the tumor [15]. However, tumorlet metastasis in the omentum suggests undetermined biological behavior.

**Table 1:** Immunohistochemical characteristics

Ref.	β-catenin	CD10	CyclinD1	WT-1	CD99	vimentin	Calretinin	inhibin-α	AR	ER	PR	AE1/AE3	CK7	Syn	CD56	SALL4	E-cadherin	SMA	CD34
[1, 3]	15/15	16/16	15/15	15/15	2/15	16/16	1/16 W	1/16 W	0/0	1/15F	0/15	0/0	0/0	6/15	2/15	0/0	0/15	0/0	0/0
[2]	1/1	1/1	0/0	0/0	1/1	1/1	0/0	0/1	0/0	0/0	0/0	0/1	0/1	0/1	0/1	0/0	0/0	0/0	0/0
[6]	6/6	6/6	0/0	0/6	3/5	6/6	0/6	0/6	0/0	0/0	2/5F	0/0	0/0	0/0	1/5F	0/0	0/5	0/0	0/0
[5]	2/2	2/2	0/0	2/2	0/2	2/2	0/2	0/2	0/0	0/2	0/2	1/2	0/2	0/2	0/2	0/2	0/0	0/2	0/2
[7]	1/1	1/1	0/0	1/1	0/0	1/1	0/1	0/1	0/0	0/0	0/0	0/0	0/0	0/0	0/0	0/0	0/1	0/0	0/0
[8]	2/2	2/2	2/2	2/2	0/2	2/2	0/2	0/2	0/2	0/2	0/2	0/0	0/0	0/2	0/2	0/2	0/2	0/2	0/2
[9]	2/2	2/2	0/0	0/0	2/2	2/2	0/0	0/0	0/0	0/0	0/0	0/0	0/0	0/0	0/0	0/0	0/0	0/0	0/0
[10]	1/1	1/1	0/0	1/1	0/0	1/1	1/1F	1/1F	0/0	0/0	0/0	0/1	0/0	0/0	0/0	0/0	0/0	0/0	0/0
[11]	1/1	1/1	0/0	0/0	0/0	1/1	1/1 + W	0/1	0/0	0/0	0/0	0/1	0/0	0/0	0/0	0/0	0/0	0/0	0/0
[12]	4/4	3/3	4/4	4/4	1/1	0/0	0/4	0/4	2/2	0/3	0/2	0/0	0/1	0/0	2/2F	0/0	0/0	0/0	0/1
[13]	1/1	1/1	0/0	0/0	0/0	1/1	0/1	0/1	0/0	0/1	0/1	0/1	0/0	0/0	0/0	0/0	0/0	0/0	0/0
[14]	1/1	1/1	1/1	1/1	0/0	1/1	0/1	0/1	0/0	0/0	0/0	0/1	0/0	0/1	0/1	0/0	0/0	0/0	0/0
Total	37/37	37/37	22/22	26/26	9/28	34/34	3/35	2/36	2/2	1/23	2/27	1/7	0/4	6/21	5/28	0/4	0/23	0/2	0/5

*W* Weak expression, *F* Focal expression

Concerning the molecular mechanism of MCST,Maeda first reported a point mutation in exon 3 of CTNNB1 in two cases [5]. Given the rarity of this tumor and the limited investigation of genomic and immunohistochemical profile, we compared our results with others reported and summarized their similarities. The Genetic characteristics of 38 cases of ovarian MCST have been summarized (Table 2). According to our retrospective study, in 26 of 38 cases in which CTNNB1 mutations were detected in the original study and all cases but one exhibited nuclear and cytoplasmic β-catenin immunoreactivity [1–14],which indicates the important role of Wnt/β-catenin in the MCST. In addition, APC mutations were identified in 5 women with MCST, 4 of whom showed clinical features of FAP [2, 4, 13, 14], which explained the strong nuclear immunostaining for β-catenin, although in the absence of β-catenin mutations, further indicating that the Wnt/β-catenin/APC pathway mediated the occurrence and development of MCST. In the present study, however, an oncogenic missense mutation (c.98C > G) in *CTNNB1* was detected. Unfortunately, APC mutational status was not checked. The patient denied a family history of FAP, and no polyps were found on gastrointestinal endoscopy. Hence, we speculate that *APC* gene mutations are unlikely to be present. In 2018, McCluggage et al. clarified that ovarian MCST may be an extracolonic manifestation of FAP and that APC mutations occur in a minority of MCSTs and are mutually exclusive to CTNNB1 mutations [4]. Accordingly**,** it is also possible that alterations in the other genes involved in the Wnt/β-catenin pathway are involved in its tumorigenesis. It is unclear whether there is a common morphology of MCST in different genetic backgrounds [16]. Therefore, it is beneficial for all females with MCST to be evaluated for FAP, including an assessment of different genes involved in the Wnt/β-catenin signaling pathway.

**Table 2:** Genetic characteristics

References	Case	CTNNB1 Mutation	APC Mutation	Comorbid FAP	Follow up time (m)
Maeda et al. [5]	1	c.98C > G p.S33C	NK	NK	14
	2	c.98C > G p.S33C	NK	NK	4
Irving et al. [3]	3	c. 95A > T, p.D32V	–	NK	NK
	4	c. 104 T > G, p.I35S	–	NK	NK
	5	c. 95A > T, p.D32V	–	NK	NK
	6	c.110C > G, p.S37C	–	NK	NK
	7	c. 109 T > C, p.S37P	–	NK	NK
	8	c. 110C > G, p.S37C c. 95A > T, p.D32V	–	NK	NK
	9	c. 101G > A, p.G34E	–	NK	NK
	10	c. 95A > T,p.D32V			NK
	11	–	c. 1620_1621insA, p.Q541Tfs*19	NK	NK
	12	c.98C > G, p.S33C	–		NK
	13	–	–		NK
	14	c. 101G > A, p.G34E	–		NK
	15	–	c.1257delC, p.T419fs c.1449 T > A, p.C483*		NK
	16	NK	NK		NK
	17	–	–		NK
M Yang et al. [11]	18	NK	NK	NK	NK
Y N et al. [7]	19	c.97 T > C p.S33P	NK	NK	NK
S H Lee et al. [13]	20	–	c.2376_2378delGCAinsCC, (p.lys792Asnfs*28). c.3796G > A, p.D1266N c.1540delG, (p.Ala514 Profs*9).	Y	NK
Bi et al. [6]	21	c.122C > T p.T41I	NK	NK	60
	22	Wild-type	NK	NK	18
	23	c.110C > G p.S37C	NK	NK	7
	24	c.101G > A p.G34E	NK	NK	NK
	25	c.97 T > C P.S33P,	NK	NK	59
	26	Wild-type	NK	NK	2
Podduturi et al. [10]	27	c.101 G > A, p.G34E	NK	NK	NK
J H Lee et al. [9]	28	c.98C > G; p.S33C	NK	NK	NK
	29	c.98C > G; p.S33C	NK	NK	NK
K Na et al. [8]	30	c.122C > T p.T41I	NK	NK	57
	31	c.88_99delTACCTGGACTCT p.Y30_S33del	NK	NK	20
W G McCluggage et al. [12]	32	c.100G > A,p.G34R	NK	NK	NK
	33	c.98C > G,p.S33C	NK	NK	NK
	34	wid-type	NK	NK	NK
	35	c.97 T > G,pS33A	NK	NK	NK
C Liu et al. [14]	36	–	Intron 6,c.730-1G > T	Y	NK
Y. Zhang et al. [2]	37	–	c.1590C > T, p.G530E	Y	108
Man et al. (this case)	38	c.98C > G,p.S33C	–	N	19

*A/Ala* Alanine, *S* Serine, *C* Cysteine, *D* Aspartic acid, *V* Valine, *I* Isoleucine, *P/Pro* Proline, *G* Glycine, *H* Histamine, *lys* Lysine, *L* Leucine, *Y* Tyrosine, *E* Glutamic acid, *T* Thorenine, *R* Arginine, *Q* Glutamine, *Asn* Asparagines, *del* Deletion, *ins* Insertion, *fs* Frame shift, *NK* Not known, *N* No, *Y* Yes, *FAP* Familial adenomatous polyposis

The behavior of MCST is not well known because of limited case reports and follow-ups, but the available information suggests that MCST is likely benign. The most unique aspect of the present case revealed a 2-mm-diameter tumorlet in the omentum. Both morphology and immunophenotype are identical to the primary ovarian MCST, which indicated that MCST is not a purely benign ovarian tumor, as previously believed. Previous studies have elucidated the crucial role of Wnt/β-catenin in the MCST. β-Catenin-mediated migration and adhesion is linked on the one hand to stimulating the expression of protooncogenes due to its nuclear accumulation and on the other to E-cadherin stabilization [17]. The E-cadherin/β-catenin complex affects cell adhesion and may regulate cancer invasion and seeding metastasis. In the current case, mutation of β-catenin may affect cell adhesion, which results in the detachment of tumor cells and causes omental deposits. On the basis of these pathological findings and molecular alterations, we assume that MCST more likely belongs to an underrecognized tumor of undetermined potential.

Not much is known regarding the biophysical behavior of MCST because of its rarity. Here, we present a rare case of ovarian MCST with omental metastasis, which alerts us to the undetermined potential, and even the malignant biological behavior, of MCST. More cases and molecular studies will be necessary to further warrant this speculation.

## Supplementary Information

**Additional file 1.**

## Data Availability

All datasets generated for this study are included in the manuscript.

## Abbreviation

MCST: Microcystic stromal tumor
