# Supplementary figures and images for: Ovarian microcystic stromal tumor with omental metastasis: the first case report and literature review
Source: J Ovarian Res. 2021 May 27;14:73. doi: 10.1186/s13048-021-00812-1 (PMC8157402; doi:10.1186/s13048-021-00812-1)

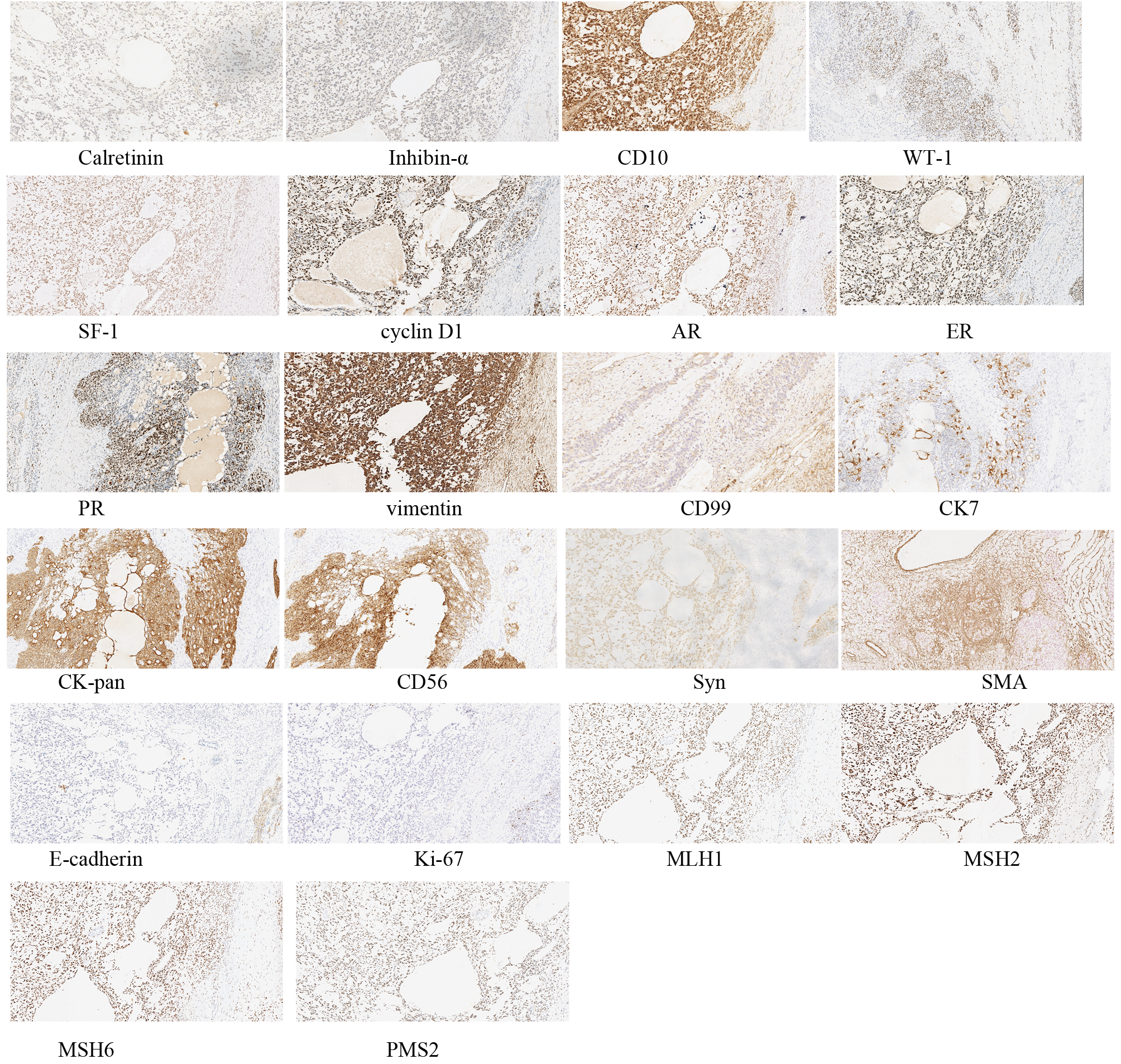

Supplement: Supplementary file 1 — Additional file 1. [file 13048_2021_812_MOESM1_ESM.tif]
